# 511. Activity of Sulbactam-durlobactam and Standard-of-Care Antibiotics *against Acinetobacter baumannii-calcoaceticus* complex isolates acquired from Hospitalized Patients in the United States (2023 – 2024)

**DOI:** 10.1093/ofid/ofae631.163

**Published:** 2025-01-29

**Authors:** Ecem Buyukyanbolu, Jill Argotsinger, Eric T Beck, Robin R Chamberland, Andrew E Clark, Philip Gialanella, Matthew Loeb, Amy Sears, Amanda Harrington, Romney Humphries, Wesley D Kufel, Scott W Riddell, A Brian Mochon, Christine A Vu, Lilian M Abbo, Octavio Martinez, Jamie Marino, Lars Westblade, David P Nicolau, Tomefa E Asempa

**Affiliations:** Hartford Hospital, Hartford, Connecticut; Advocate Lutheran General Hospital, Park Ridge, Illinois; Department of Microbiology, ACL Laboratories, West Allis, Wisconsin; Saint Louis University School of Medicine, St. Louis, Missouri; University of Texas Southwestern Medical Center, Dallas, Texas; Montefiore Medical Center, Albert Einstein College of Medicine, Bronx, New York; Kansas University Medical Center, Kansas City, Kansas; Kansas University Medical Center, Kansas City, Kansas; Loyola University Chicago, Maywood, Illinois; Vanderbilt University Medical Center, Nashville, Tennessee; Binghamton University School of Pharmacy Sciences, Binghamton, NY; State University of New York Upstate Medical University, Syracuse, New York; University of Arizona College of Medicine, Phoenix, Arizona; Jackson Memorial Hospital, Miami, Florida; University of Miami Miller School of Medicine, Jackson Health System, Aventura, FL; Jackson Health System/University of Miami, Miami, FL; Weill Cornell Medicine, NY, New York; Weill Cornell Medicine, NY, New York; Hartford Hospital, Hartford, Connecticut; Hartford Hospital, Hartford, Connecticut

## Abstract

**Background:**

*Acinetobacter baumannii-calcoaceticus* complex (ABC) isolates are responsible for severe infections, such as bacteremia, healthcare-associated pneumonia, and skin and soft tissue infections. Sulbactam-durlobactam is a FDA-approved targeted β-lactam-β-lactamase inhibitor combination antibiotic developed to treat infections caused by ABC. This study aims to evaluate the *in vitro* activity of sulbactam-durlobactam and clinically utilized antibiotics against ABC strains with a predominance of carbapenem resistance.

**Table 1.**
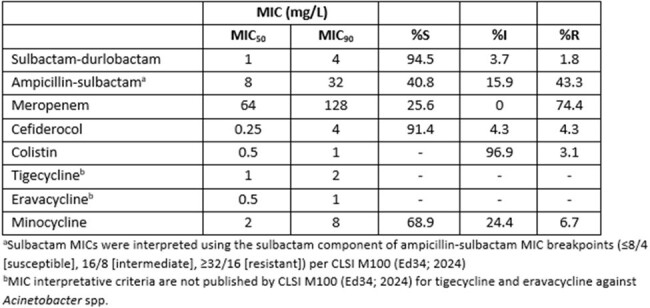
Susceptibility test results of sulbactam-durlobactam and comparator agents against Acinetobacter baumannii-calcoaceticus complex isolates

**Methods:**

164 ABC isolates in addition to de-identified data including patient location at the time of culture and culture source were submitted by 11 geographically dispersed U.S medical centers in 2023 and 2024. Susceptibility tests for sulbactam-durlobactam (durlobactam fixed concentration of 4 mg/L), and comparator agents were conducted by manual broth microdilution and interpreted according to CLSI M100Ed34 standards. Carbapenem-resistant ABC (CRAB) was defined by phenotypic resistance to meropenem (MIC ≥8 mg/L).

**Results:**

ABC isolates were primarily cultured from respiratory sources (57%), followed by blood (19%) and urine (11%). Of the 164 isolates, majority were isolated from non-ICU patients (n=102; 62%) versus ICU (n=62; 38%). The MIC_50_ and MIC_90_ values of the sulbactam component of ampicillin-sulbactam for all isolates was 8 and 32 mg/L, respectively resulting in a susceptibility rate of 40.8%. The addition of durlobactam to sulbactam decreased the MIC_50_ and MIC_90_ by 2-doubling dilutions to 1 and 4 mg/L and increased the susceptibility rate to 94.5%. The MIC_50/90_ and susceptibility rates for all other antimicrobial agents are shown in **Table 1**. Notably, a majority of isolates were CRAB (n=122; 74.4%) and among these isolates, sulbactam-durlobactam MIC_50_, MIC_90_, and %S was 2, 4 mg/L, and 92.6%, respectively.

**Conclusion:**

Sulbactam-durlobactam demonstrated potent *in vitro* activity against a contemporary challenge set of clinical ABC isolates, including carbapenem-resistant isolates among hospitalized patients in the United States. This study highlights the important role of sulbactam-durlobactam, relative to other clinically utilized agents for the treatment of infections caused by ABC isolates.

**Disclosures:**

**Amanda Harrington, PhD**, Beckman Coulter: Advisor/Consultant|bioMeriuex: Advisor/Consultant|bioMeriuex: Grant/Research Support|Bio-Rad: Advisor/Consultant|Day Zero: Advisor/Consultant|Selux Diagnostics: Grant/Research Support **Wesley D. Kufel, Pharm.D., BCPS, BCIDP**, Merck & Co.: Grant/Research Support|Shionogi, Inc: Grant/Research Support **Lars Westblade, PhD**, Accelerate Diagnostics, Inc: Grant/Research Support|bioMerieux, Inc: Grant/Research Support|Element Materials Technology: Grant/Research Support|Hardy Diagnostics: Grant/Research Support|Roche Molecular Systems, Inc.: Advisor/Consultant|Roche Molecular Systems, Inc.: Grant/Research Support|Selux Diagnostics, Inc.: Grant/Research Support|Shionogi, Inc: Advisor/Consultant|Talis Biomedical: Advisor/Consultant **David P. Nicolau, PharmD**, CARB-X: Grant/Research Support|Innoviva: Grant/Research Support|Innoviva: Honoraria|Merck: Advisor/Consultant|Merck: Grant/Research Support|Merck: Honoraria|Pfizer: Advisor/Consultant|Pfizer: Grant/Research Support|Pfizer: Honoraria|Shionogi: Advisor/Consultant|Shionogi: Grant/Research Support|Shionogi: Honoraria|Venatorx: Grant/Research Support **Tomefa E. Asempa, PharmD**, FDA/CDER: Grant/Research Support|Paratek: Grant/Research Support|Shionogi: Grant/Research Support|Spero: Grant/Research Support|VenatoRx: Grant/Research Support

